# LTE: Does caffeine truly raise muscle carnitine in humans?

**DOI:** 10.14814/phy2.15736

**Published:** 2023-08-31

**Authors:** Dumitru Constantin‐Teodosiu

**Affiliations:** ^1^ School of Life Sciences, Division of Physiology, Pharmacology and Neuroscience, Queen's Medical Centre University of Nottingham Medical School Nottingham UK

A recent study led by FB Stephens (Wall et al., 2023) concluded that caffeine stimulates human muscle carnitine retention during conditions of supraphysiological plasma carnitine concentrations. Although the authors did not provide any other evidence of carnitine data except that in plasma, they anticipated that carnitine with caffeine may represent a novel muscle carnitine loading strategy aimed to improve exercise performance or fat oxidation in type II diabetic patients.

On this background, there are, however, theoretical and numeric concerns, which might not support the authors' views.

The supraphysiological plasma carnitine concentrations were achieved by an initial carnitine oral ingestion of 6 mg/kg body mass (bm), followed by two intravenous carnitine provisions (one bolus of 15 mg/kg bm sustained over 10 min and one continuous infusion of 10 mg/kg bm/h over 290 min). After normalization to the average bm of the participants recruited for the study (68 kg bm), the average total carnitine provided to each subject mounted to 4698 mg carnitine (6 mg/kg bm × 68 kg bm = 410 mg, 15 mg/kg bm × 68 kg bm = 1002 mg, and 10 mg/kg bm/h × 68 kg bm × 4.83 h [290 min/60] = 3286 mg).

If we look at Figure 1b of the study reproduced here (Wall et al., 2023), it can be noticed that the steady state of plasma carnitine concentration (~350 μm/L) was reached after 30 min of carnitine infusion. At this point, all subjects received 1636 mg (410 mg + 1002 mg + 10 mg/kg/h × 68 kg × 0.33 h [20 min]) of carnitine. Since no muscle carnitine accretion would have occurred in the carnitine‐only group (Wall et al., 2023), the same event should have occurred in the carnitine + caffeine group too as the slopes of the rise in plasma carnitine were identical in both groups (Figure 1b). Therefore, the carnitine bioavailability in any group at the start of the steady‐state phase would have been 156 mg (350 μmol/L × 0.001 [μmol to mmol] × 2.75 L plasma volume [5 L blood × 0.055] × 162 mg/mmol [carnitine molecular weight]) or 9.5% of the carnitine made available (156/1636). This assessment would be in line with previously recorded values of carnitine bioavailability (5%–18%; Evans & Fornasini, 2003). It is firmly believed that this low carnitine bioavailability is attributable to the inability of the kidney to reabsorb carnitine when the threshold concentration for tubular reabsorption (about 40–60 μmol/L, which is similar to the present endogenous plasma carnitine level; Figure 1b) has passed this value (Evans & Fornasini, 2003).

**FIGURE 1:**
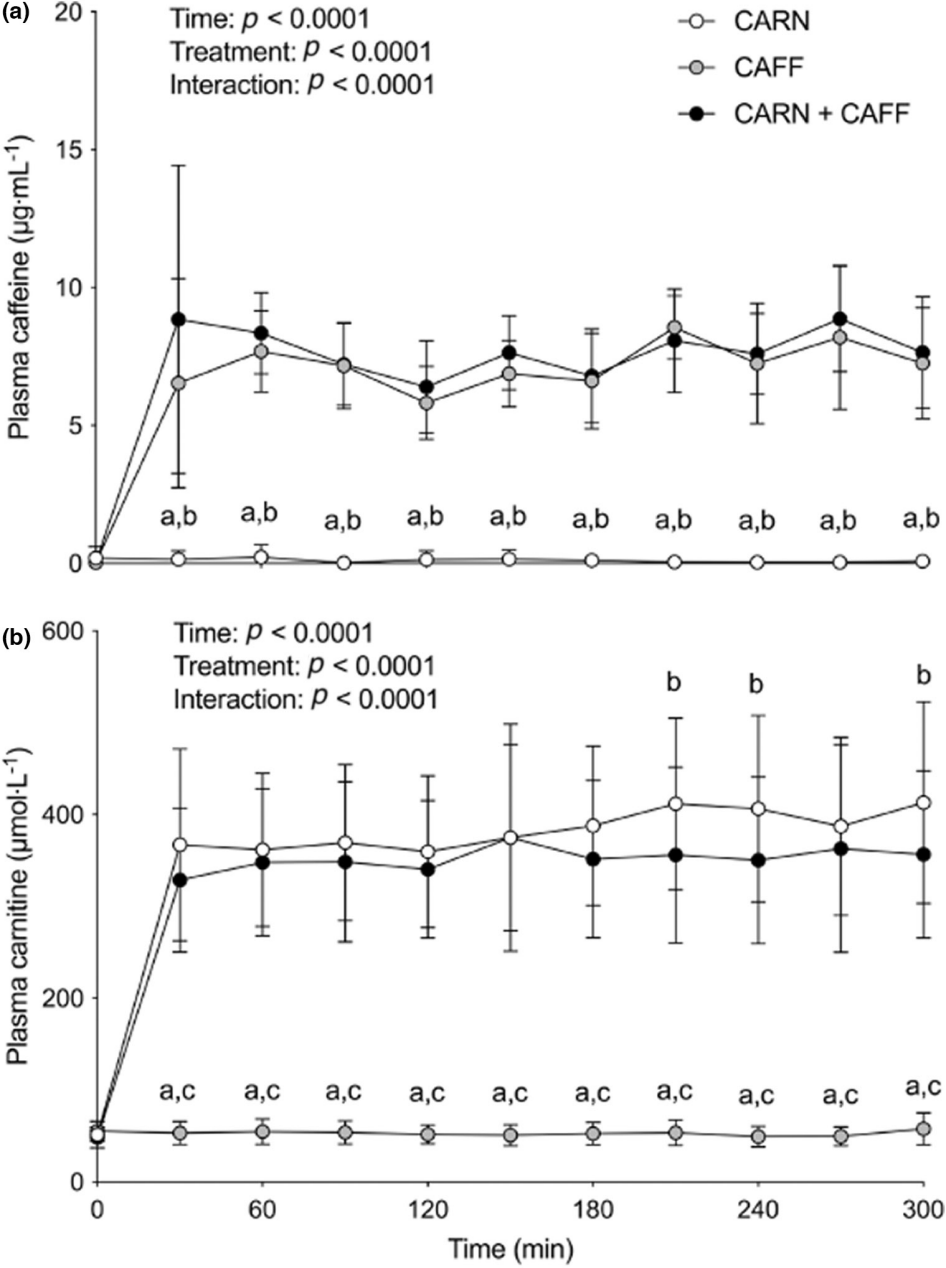
Plasma caffeine (a) and free carnitine (b) concentrations at baseline (i.e., 0 min) and throughout 5 h primed and continuous intravenous infusions of saline (CAFF) or l‐carnitine (CARN and CARN + CAFF), and ingestion of placebo (CARN) or 9 mg/kg body mass (bm) caffeine (consumed as 6 mg/kg bm at baseline and 1 mg/kg bm after 120, 180, and 240 min) (CAFF and CARN + CAFF) administered in a randomized, counterbalanced, crossover, double‐blind fashion in six healthy young adults. Data (*n* = 6) are presented as mean + SD and analysed with repeated measures two‐way ANOVAs (time and treatment; main effects depicted) with Bonferroni post hoc tests where “a” denotes a significant difference between corresponding values of CARN and CAFF (*p* < 0.0001), “b” denotes a significant difference (*p* < 0.0001 in a and *p* < 0.01 in b) between corresponding values of CARN and CARN + CAFF, and “c” denotes a significant difference (*p* < 0.0001) between corresponding values of CAFF and CARN + CAFF. Reproduced from Wall et al., 2023.

Figure 1b also shows that over the last 90 min of carnitine infusion, a relative difference in plasma carnitine concentration across the groups (~Δ 55 μmol/L lower in the carnitine + caffeine group) became apparent. During the new conditions of steady‐state and similar rates of carnitine provision, this difference could reflect a one‐off additional 25 mg of free carnitine (Δ 55 μmol/L × 0.001 [μmol to mmol] × 2.75 L plasma volume [5 L blood × 0.55] × 162 mg/mmol [carnitine molecular weight]) being cleared from the plasma of the subjects on carnitine + caffeine. According to the authors' interpretation, this additional withdrawal of carnitine from plasma would hint at a greater skeletal muscle carnitine uptake in the carnitine + caffeine group. However, this minute amount of carnitine (25 mg) should also be viewed from the perspective of the amount of supplemented carnitine (4698 mg) and that of the whole‐body carnitine content of 16,184 mg (3.7 mmol carnitine/kg wet muscle [Constantin‐Teodosiu et al., 1992] × 27 kg wet muscles [68 kg bm × 0.4] × 162 mg/mmol) before assigning any biological importance. More importantly, however, is the consideration that since carnitine is mainly eliminated from the body via urinary excretion, then this small plasma carnitine difference recorded in the last 90 min of the infusion would have been most likely accounted for by a greater renal carnitine clearance in the caffeine‐supplemented group, as caffeine, which is a strong diuretic compound, would have certainly increased the urinary output.

Based on the finding that intravenous provision with carnitine alone does not increase muscle carnitine accretion (Wall et al., 2023) and on the above‐re‐evaluated data, it appears that the basis for carnitine with caffeine being able to increase muscle carnitine levels, and thereby manipulation of muscle metabolism and exercise performance, is uncertain.

Interestingly, caffeine increases the sympathetic nervous system responses, and thereby the release of norepinephrine, epinephrine, and cortisol. These hormonal changes increase hepatic glucose production and lipolysis, which collectively lower, rather than increase, the overall insulin sensitivity (Lee et al., 2020). Another detail that would undo the recommendation of such protocol is the observation that obese people who might have or have not developed type II diabetes, or healthy subjects who have consumed a high‐fat diet for several days, collectively display higher muscle carnitine stores than lean subjects on normal diets (Constantin‐Teodosiu et al., 2019). Therefore, the authors' proposed long‐term use of carnitine supplementation as an aid to improve fat oxidation in type II diabetes also seems to lack provision.

## CONFLICT OF INTEREST STATEMENT

The author would like to disclose the following interests that may directly or indirectly be viewed as being related to the work submitted in the Letter to Editor.The author is a co‐applicant for the USA patent US9662344B2 “Carnitine retention”, which relates to carnitine accretion in animal and human tissue using a carnitine form along an agent to increase Na+/K+ ATPase pump, and/or to increase the activity of a carnitine transport protein, and/or increase blood/plasma insulin level.The author would also like to highlight two publications related to plasma carnitine clearance co‐authored with Dr. FB Stephens, who is also the corresponding author of the publication the present Letter to Editor comments on:Stephens, F.B., Constantin‐Teodosiu, D., Laithwaite, D., Simpson, E.J., & Greenhaff, P.L. (2006). Insulin stimulates L‐carnitine accumulation in human skeletal muscle. The FASEB Journal, 20, 377–379.Stephens, F. B., Constantin‐Teodosiu, D., Laithwaite, D., Simpson, E.J., & Greenhaff, P.L. (2007). A threshold exists or the stimulatory effect of insulin on plasma L‐carnitine clearance in humans. American Journal of Physiology. Endocrinology and Metabolism, 292, E637–E641.

## References

[phy215736-bib-0001] Constantin‐Teodosiu, D. , Cederblad, G. , Bergstrom, M. , & Greenhaff, P. L. (2019). Maximal‐intensity exercise does not fully restore muscle pyruvate dehydrogenase complex activation after 3 days of high‐fat dietary intake. Clinical Nutrition, 38, 948–953. 29459213 10.1016/j.clnu.2018.02.001

[phy215736-bib-0002] Constantin‐Teodosiu, D. , Cederblad, G. , & Hultman, E. (1992). PDC activity and acetyl group accumulation in skeletal muscle during prolonged exercise. Journal of Applied Physiology, 1985(73), 2403–2407. 10.1152/jappl.1992.73.6.2403 1490950

[phy215736-bib-0003] Evans, A. M. , & Fornasini, G. (2003). Pharmacokinetics of L‐carnitine. Clinical Pharmacokinetics, 42, 941–967. 12908852 10.2165/00003088-200342110-00002

[phy215736-bib-0004] Lee, S. , Min, J. Y. , & Min, K. B. (2020). Caffeine and caffeine metabolites in relation to insulin resistance and Beta cell function in U.S adults. Nutrients, 12, 1783. 32549382 10.3390/nu12061783 PMC7353167

[phy215736-bib-0005] Wall, B. T. , Machin, D. , Dunlop, M. V. , & Stephens, F. B. (2023). Caffeine ingestion stimulates plasma carnitine clearance in humans. Physiological Reports, 11, e15615. 36806708 10.14814/phy2.15615 PMC9938004

